# Association of SMAD4 mutation with patient demographics, tumor characteristics, and clinical outcomes in colorectal cancer

**DOI:** 10.1371/journal.pone.0173345

**Published:** 2017-03-07

**Authors:** Amir Mehrvarz Sarshekeh, Shailesh Advani, Michael J. Overman, Ganiraju Manyam, Bryan K. Kee, David R. Fogelman, Arvind Dasari, Kanwal Raghav, Eduardo Vilar, Shanequa Manuel, Imad Shureiqi, Robert A. Wolff, Keyur P. Patel, Raja Luthra, Kenna Shaw, Cathy Eng, Dipen M. Maru, Mark J. Routbort, Funda Meric-Bernstam, Scott Kopetz

**Affiliations:** Department of Gastrointestinal Medical Oncology, The University of Texas MD Anderson Cancer Center, Houston, Texas, United States of America; University of Nebraska Medical Center, UNITED STATES

## Abstract

SMAD4 is an essential mediator in the transforming growth factor-β pathway. Sporadic mutations of SMAD4 are present in 2.1–20.0% of colorectal cancers (CRCs) but data are limited. In this study, we aimed to evaluate clinicopathologic characteristics, prognosis, and clinical outcome associated with this mutation in CRC cases. Data for patients with metastatic or unresectable CRC who underwent genotyping for SMAD4 mutation and received treatment at The University of Texas MD Anderson Cancer Center from 2000 to 2014 were reviewed. Their tumors were sequenced using a hotspot panel predicted to cover 80% of the reported SMAD4 mutations, and further targeted resequencing that included full-length SMAD4 was performed on mutated tumors using a HiSeq sequencing system. Using The Cancer Genome Atlas data on CRC, the characteristics of SMAD4 and transforming growth factor-β pathway mutations were evaluated according to different consensus molecular subtypes of CRC. Among 734 patients with CRC, 90 (12%) had SMAD4 mutations according to hotspot testing. SMAD4 mutation was associated with colon cancer more so than with rectal cancer (odds ratio 2.85; p<0.001), female sex (odds ratio 1.71; p = 0.02), and shorter overall survival than in wild-type SMAD4 cases (median, 29 months versus 56 months; hazard ratio 2.08; p<0.001 [log-rank test]). SMAD4 mutation was not associated with age, stage at presentation, colonic location, distant metastasis, or tumor grade. A subset of patients with metastatic CRC (n = 44) wild-type for KRAS, NRAS, and BRAF who received anti-epidermal growth factor receptor therapy with mutated SMAD4 (n = 13) had shorter progression-free survival duration than did patients wild-type for SMAD4 (n = 31) (median, 111 days versus 180 days; p = 0.003 [log-rank test]). Full-length sequencing confirmed that missense mutations at R361 and P356 in the MH2 domain were the most common SMAD4 alterations. In The Cancer Genome Atlas data, SMAD4 mutation frequently occurred with KRAS, NRAS, and BRAF mutations and was more common in patients with the consensus molecular subtype 3 of CRC than in those with the other 3 subtypes. This is one of the largest retrospective studies to date characterizing SMAD4 mutations in CRC patients and demonstrates the prognostic role and lack of response of CRC to anti-epidermal growth factor receptor therapy. Further studies are required to validate these findings and the role of SMAD4 mutation in CRC.

## Introduction

Colorectal cancer (CRC) is the third most commonly diagnosed cancer in the United States, and researchers estimated that about 150,000 new cases of CRC would be diagnosed in 2016. Although screening practices have led to a decline in CRC mortality rates since the 1990s, researchers estimated that 49,190 CRC-related deaths would occur in the United States in 2016 [1]. The prognosis for CRC is widely variable, and about 20% of cases are metastatic at the time of presentation.

Over the past few decades, advances in molecular biology have helped identify and characterize genes and molecular pathways involved in carcinogenesis, disease progression, and resistance to treatment in CRC patients. Tumor genotyping and sequencing technology—now standard practice—assist clinicians in predicting disease behavior, prognosis, and treatment response, but more prognostic markers are required to further personalize treatment plans and differentiate among various subgroups of patients. Therefore, the identification of new markers remains essential to improving treatment outcomes and survival in CRC patients [2, 3].

The transforming growth factor (TGF)-β signaling pathway has a critical role in CRC progression. This pathway is naturally involved in many biologic cellular processes such as cell proliferation, differentiation, apoptosis, and extracellular matrix production [4]. Alteration of TGF-β has pivotal roles in carcinogenesis and cancer progression that are paradoxical. In the early stages of cancer development, activation of TGF-β is associated with tumor suppression [5], but in the more advanced stages, TGF-β is believed to promote metastasis, angiogenesis, and epithelial-to-mesenchymal transition [6, 7]. TGF-β signaling is initiated by the binding of TGF-β ligands to TGF-β transmembrane protein kinase receptors. Upon activation of TGF-β receptors (including TGF-β receptors 1 and 2), phosphorylation of the receptor-activated SMADs and the SMAD protein family members (including SMAD2 and SMAD3) occurs, which enables SMAD2 and SMAD3 to bind to SMAD4 [8, 9]. The resulting complex relocates into the nucleus and regulates transcription of TGF-β–related genes [10–13].

SMAD proteins are essential mediators of the TGF-β signaling pathway. These proteins are highly homologous and harbor 2 conserved domains known as the N-terminal Mad homology domain-1 and C-terminal Mad homology domain-2 (MH2) [14]. The N-terminal Mad homology domain-1 is involved in negative regulation of MH2, nuclear import, and the binding of the DNA transcriptional co-regulator [14, 15]. The MH2 domain is involved in SMAD protein homo- and hetero-oligomerization, cytoplasmic anchoring, and transcription of target genes [10–12, 16, 17]. Among this protein family, SMAD4 (localized to band 18q21) has a central role as a common downstream regulator and tumor suppressor gene [18, 19]. In previous studies, the loss of SMAD4 function was an independent prognostic factor for decreased recurrence-free and overall survival (OS) in CRC patients, particularly those with advanced-stage disease [20–23]. On the other hand, higher levels of SMAD4 expression have been associated with better OS and disease-free survival in CRC patients [21]. In addition, inactivation of SMAD4 has rendered cells resistant to TGF-β1 –induced growth inhibition [24]. Sporadic mutation of SMAD4 has been reported in 2.1–20.0% of CRC cases [25–30]. The occurrence of SMAD4 mutation in CRC is considered a late event and usually happens in combination with other alterations [27, 31]. In a study performed on primary and metastatic tumor pairs, SMAD4 mutation was one of the frequent novel mutations in metastases; the finding that suggests SMAD4 is involved in clonal divergence [32]. Despite the essential role of this protein in TGF-β signaling pathway, data regarding clinicopathologic features, treatment response, and outcome in patients with SMAD4-mutated tumors is limited. Therefore, the purpose of the present study was to evaluate characteristics, survival, and treatment response associated with SMAD4 mutation, and identify characteristics of SMAD4 and TGF-β pathway mutations in CRC cases in The Cancer Genome Atlas (TCGA) database cases.

## Materials and methods

### Patients

Patients with metastatic or locally advanced unresectable CRC who received treatment at The University of Texas MD Anderson Cancer Center from 2000 to 2014 and whose tumors were genotyped for SMAD4 mutations, were included in this retrospective study. Tumors were sequenced using a hotspot panel (Ion Torrent, Life Technologies, Carlsbad, CA, USA) predicted to cover 80% of the reported mutations of SMAD4. Further targeted resequencing that included full-length SMAD4 was performed with a focus on mutated tumors using a HiSeq sequencing system (Illumina, San Diego, CA, USA) with full exome coverage to an average depth of 800. Patients were categorized based on their SMAD4 mutational status as SMAD4-mutated or SMAD4 wild-type.

Clinicopathologic information on the study patients was obtained from the CRC registry database at MD Anderson. The collected information included age at the time of presentation (<65 years versus ≥65 years), sex, race, site of primary tumor (colon or rectum), tumor grade (well, moderately, or poorly differentiated), presence of distant metastasis at the time of presentation, date of diagnosis, date of last contact with MDACC, and vital status. Follow-up information on living patients was collected through December 2015. The average follow-up duration for all patients was 50 months. For a subset of patients who had available data on anti-epidermal growth factor receptor (EGFR) treatment, treatment response was evaluated according to SMAD4 mutational status. Patients with known SMAD4 mutational statuses who were wild-type for KRAS, NRAS, and BRAF genes and experienced progression of disease while receiving anti-EGFR treatment were included in treatment response analysis. For these patients, the dates of initiation and termination of anti-EGFR treatment and the anti-EGFR agent (cetuximab or panitumumab) used were retrospectively reviewed. All patients signed informed written consent and the study protocol was approved by the MD Anderson Institutional Review Board.

### Mutational status analysis

Consensus molecular subtypes (CMSs) of CRC were established by a large-scale analytical study interconnecting 6 independent CRC classification systems [33]. Each of the 4 molecular subtypes according to gene expression has very unique molecular characteristics representing the mesenchymal (CMS4), metabolic (CMS3), canonical (CMS2), and microsatellite instability/immune (CMS1) phenotypes. In the present study, CMS classifications of TCGA CRC samples were used to determine the frequency distribution for SMAD4 and TGF-β signaling pathway mutations across the molecular subtypes [34]. The mutation information for the TCGA CRC-sequenced samples (n = 224) within the members of the TGF-β signaling pathway was obtained from the cBioPortal for Cancer Genomics database [25, 35, 36]. In this data set, only samples classified into one of the molecular subtypes (n = 171) were used to assess mutation frequency. The frequency of the mutations in the TGF-β signaling pathway was quantified using the R-language ComplexHeatmap software package. The genetic alterations of KRAS, NRAS, and BRAF in CRC cells in TCGA CRC patient data (n = 220) were reviewed, and the co-occurrence of SMAD4 mutation and these mutations was quantified.

### Statistical analysis

Patient demographic and clinical characteristics were compared according to SMAD4 mutational status. Comparisons across groups were done using chi-square tests and the Fisher’s exact test. Logistic regression analysis was performed to calculate the odds ratio for development of SMAD4-positive tumors according to various demographic and clinical characteristics. The Fisher exact test was also used to determine the association of SMAD4 and TGF-β protein mutations across the CMS classifications.

Also, survival analysis was performed to determine whether the SMAD4 mutation status plays a role in clinical outcomes of metastatic CRC. OS was defined as the time from diagnosis of CRC to death owing to any cause and analyzed using the Kaplan-Meier method. Log-rank testing was used to compare survival curves across mutational statuses. Univariate and multivariate Cox regression analyses were performed to determine the association of various factors with OS in metastatic CRC cases. Variables, which were associated with poor OS in the univariate analysis, were included in the multivariate Cox regression analysis. All p values were 2-sided, and statistical significance was set at p<0.05. Statistical analyses were performed using the Stata (version 13.0) and SPSS Windows (version 16.0) software programs (SPSS Inc, Chicago, IL, USA).

## Results

### Mutation frequency and population characteristics

**Fig 1:**
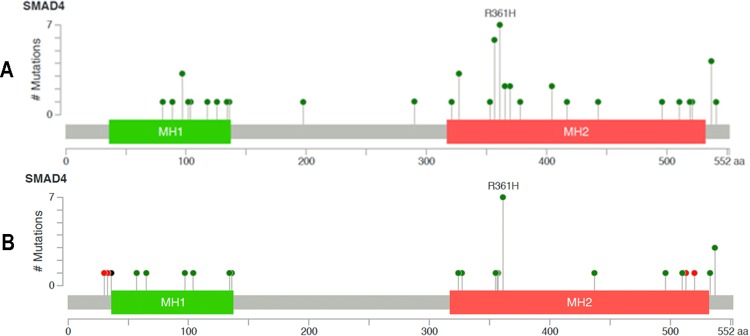
The prevalence and spectrum of SMAD4 mutations in the MD Anderson study patients and TCGA data. (A) The prevalence and spectrum of SMAD4 mutations in the study patients who underwent full-length sequencing (n = 49). (B) The prevalence and spectrum of SMAD4 mutations in TCGA data (n = 220 patients).

Of a total of 734 patients, 90 (12%) had SMAD4 mutations identified by hotspot testing. Full-length sequencing was performed in 49 mutated tumors in the study population. Missense mutations at R361 and P356 in the MH2 domain were the most common mutations of the SMAD4 gene (Fig 1).

### Association of clinicopathologic features and OS with mutational status

The patients’ demographic and tumor characteristics are listed in Table 1. The majority of the patients were male (n = 396 [54%]), and the median age at the time of diagnosis was 52 years. Overall, most patients had metastatic disease at the time of presentation (n = 600 [82%]).

**Table 1:** Demographic and tumor characteristics of CRC patients according to SMAD4 mutation status.

	Wild-type SMAD4 (n = 644) (%)	Mutated SMAD4 (n = 90) (%)	P
**Sex**			0.020
• Female	286 (44)	52 (58)	
• Male	358 (56)	38 (42)	
**Race/Ethnicity**			0.062*
• White	478 (4)	71 (79)	
• Black	63 (10)	9 (10)	
• Hispanic	57 (9)	7 (8)	
• Other	46 (7)	3 (3)	
**Age at the time of presentation**			0.320
• <65 years	561 (87)	75 (83)	
• ≥65 years	83 (13)	15 (17)	
**Site of primary tumor**			0.001
• Colon	410 (64)	75 (83)	
• Rectum	234 (36)	15 (17)	
**Disease stage at the time of presentation (available data: n = 691)**			0.260*
• Stage II	14 (2)	0	
• Stage III	71 (11)	6 (7)	
• Stage IV	526 (82)	74 (82)	
**Presence of distant metastasis (available data: n = 691)**			0.110
• Yes	526 (82)	74 (82)	
• No	85 (13)	6 (7)	
**Tumor grade (available data: n = 534)**			0.828
• Well to moderately differentiated	357 (76)	50 (79)	
• Poorly differentiated	114 (24)	13 (20)	

* F-test.

We compared the demographic and clinical characteristics of our patients according to SMAD4 mutational status and found that SMAD4 mutation was associated with sex and tumor site. In univariate analysis, female patients were more likely to harbor SMAD4 mutations in their tumors than were male patients (odds ratio, 1.71, [95% confidence interval (CI) 1.07–2.74]; p = 0.02). Also, compared with white patients, other racial groups tended to have lower rates of SMAD4 mutation, though this difference was not significant (p = 0.062). Furthermore, SMAD4-mutant tumors were more likely to be located in the colon than in the rectum (odds ratio, 2.85 [95% CI 1.55–5.31]; p<0.001).

**Fig 2:**
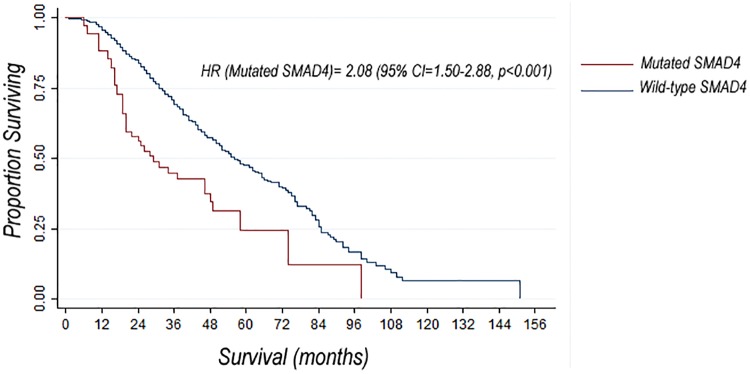
Comparison of survival curves in patients with metastatic CRC according to SMAD4 mutation status. Median OS durations of 29 months (95% CI, 20–48 months) and 56 months (95% CI, 51–63 months) were observed in patients with mutated and wild-type SMAD4, respectively.

The median OS duration for all patients in the study was 62 months. We compared OS in the subset of patients with metastatic disease (n = 600). When compared with patients wild type for SMAD4, those with SMAD4 mutations had shorter OS duration from date of diagnosis (median, 29 months [95% CI, 20-48months] versus 56 months [95% CI, 51–63 months]; p<0.001) (Fig 2). We also performed univariate and multivariate Cox regression analyses of the association of SMAD4 mutation status and other factors with OS in patients with metastatic disease. In univariate analysis, SMAD4 mutation was associated with a poor OS (HR, 2.08 [95% CI, 1.50–2.88]; p<0.001). In addition to SMAD4 mutation status, variables associated with OS in univariate Cox regression analysis were Hispanic ethnicity and poor tumor differentiation. In multivariate Cox regression analysis, SMAD4 mutation was associated with poor OS (HR, 1.39 [95% CI, 0.93–2.07]; p = 0.10) after adjusting for other characteristics (Table 2).

**Table 2:** Univariate and multivariate Cox regression analyses of OS in metastatic CRC patients (n = 600).

	Univariate analysis		Multivariate analysis	
	HR (95% CI)	p	HR (95% CI)	p
**SMAD4 status**				
Wild-type	Ref		Ref	
Mutated	2.08 (1.50–2.88)	<0.001	1.39 (0.93–2.07)	0.100
**Sex**				
Female	Ref			
Male	0.98 (0.81–1.22)	0.920		
**Race/Ethnicity**				
White	Ref		Ref	
Black	1.22 (0.89–1.66)	0.220	1.3 (0.90–1.87)	0.160
Hispanic	1.45 (1.03–2.03)	0.030	1.31 (0.90–1.90)	0.170
Other	0.74 (0.50–1.16)	0.250	0.70 (0.38–1.28)	0.240
**Age at the time of presentation**				
<65 years	Ref			
≥65 years	1.08(0.81–1.46)	0.590		
**Site of primary tumor**				
Colon	Ref			
Rectum	0.88 (0.70–1.10)	0.260		
**Tumor grade (available data: n = 587)**				
Well to moderately differentiated	Ref		Ref	
Poorly differentiated	1.64 (1.25–2.14)	<0.001	1.64 (1.25–2.15)	<0.001

Ref: reference;

### Response of CRC to anti-EGFR treatment

**Fig 3:**
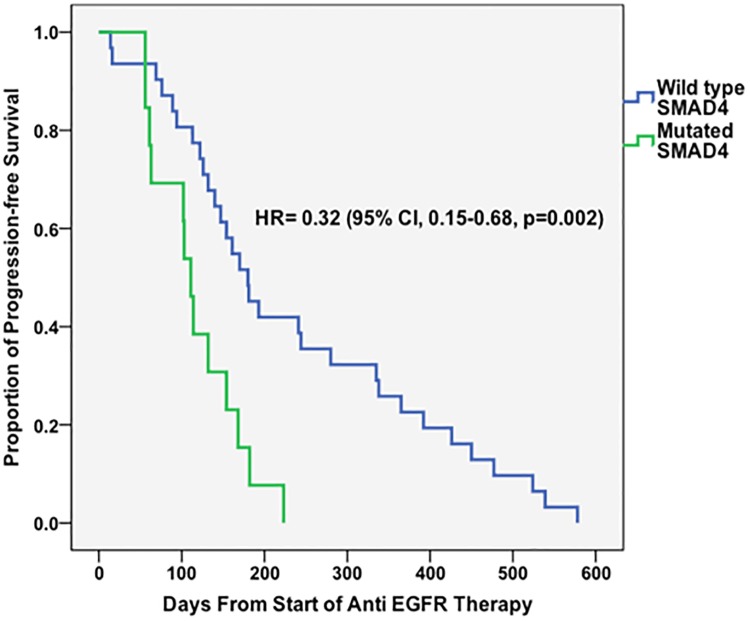
Progression-free survival curves for CRC patients who received anti-EGFR treatment according to SMAD4 mutation status. All patients were wild-type for KRAS, NRAS, and BRAF genes.

We included 44 patients (48% females [n = 21]) wild-type for the KRAS, NRAS, and BRAF genes in our treatment response analysis, 13 of whom (30%) had SMAD4 mutations. The majority of these patients (n = 26 [55%]) received line anti-EGFR treatment as third-line systemic therapy. Specifically, patients received cetuximab (n = 39 [88%]), panitumumab (n = 4 [9%]), or both (n = 1 [2%]). Majority of patients received anti-EGFR treatment in combination with irinotecan (10 [77.0%] patients with mutated SMAD4 and 23 [74.2%] patients with wild-type SMAD4). More than two thirds of the patients (n = 31 [71%]) had colonic primary tumors. Patients with SMAD4 mutations had significantly shorter progression-free survival durations than did those wild-type for SMAD4 (median, 111 days [95% CI, 96–125 days] versus 180 days [95% CI, 137–222 days]; HR, 0.32 [95% CI, 0.15–0.68]; p = 0.003). Progression-free survival curves are displayed in Fig 3.

#### Genetic analyses of TCGA data

**Fig 4:**
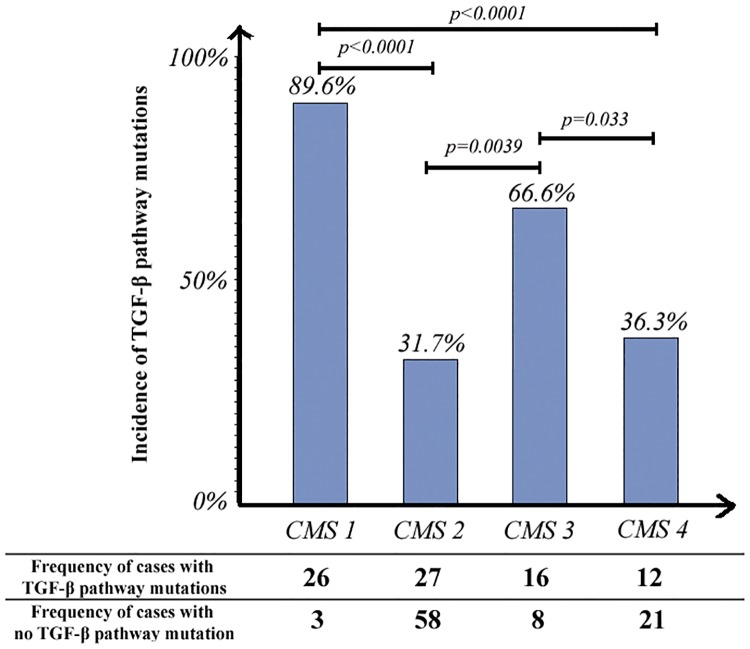
Comparison of the incidences of TGF-β pathway mutations across different molecular subtypes.

**Fig 5:**
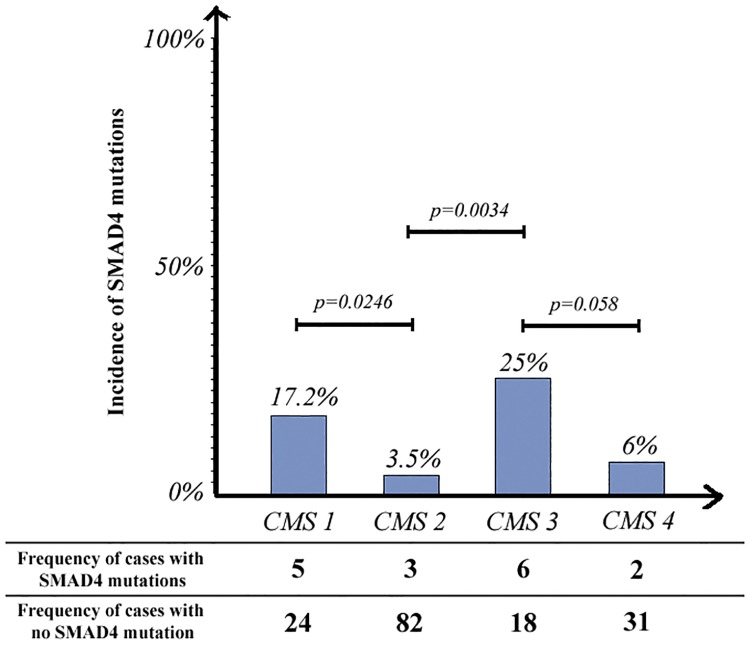
Comparison of the incidences of SMAD4 mutations across different molecular subtypes.

Figs 4 and 5 show the incidences of TGF-β pathway and SMAD4 mutations, respectively, across different molecular subtypes. SMAD4 mutations were more common with the CMS3 (metabolic) (25% mutated) than with the other 3 molecular subtypes. Two thirds of the CMS3 samples (66.6%) and approximately one third of the CMS2 (31.8%) and CMS4 (36.4%) samples had at least one mutation in the TGF-β signaling pathway (Fig 5).

**Fig 6:**
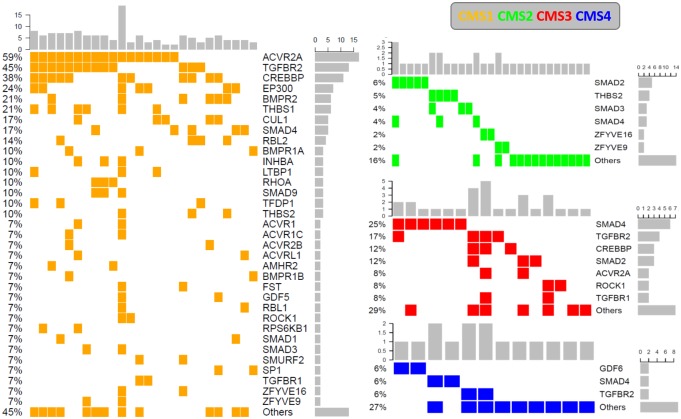
OncoPrint showing the distribution of TGF-β mutations across different molecular subtypes in TCGA CRC samples.

An OncoPrint of the mutations in the TGF-β signaling pathway across different consensus molecular subtypes in TCGA CRC cases is shown in Fig 6. In CMS3 and CMS4 samples, we observed a pattern of mutual exclusivity between SMAD4 mutations and other frequent mutations in the TGF-β signaling pathway such as TGFBR2, CREBBP, ACVR2A, SMAD2, and SMAD3 mutations, which suggest functional relation of these proteins.

**Fig 7:**
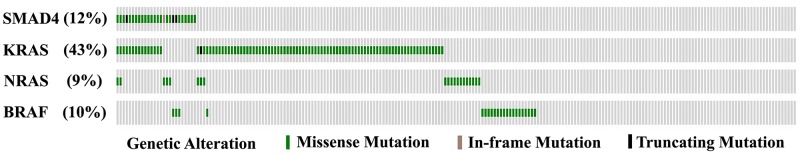
OncoPrint of genomic alterations in TCGA CRC cases (n = 220) showing mutations of SMAD4, KRAS, NRAS, and BRAF.

Also, an OncoPrint of TCGA CRC cases sequenced for SMAD4, KRAS, NRAS, and BRAF mutations is shown in Fig 7. We observed a statistically significant co-occurrence of SMAD4 mutations and these mutations in these cases (odds ratio, 3.14 [95% CI, 1.06–9.95]; p = 0.021 [chi-square test]).

## Discussion

SMAD4 protein has a pivotal role in TGF-β signaling pathway by regulating gene expression following activation of TGF-β receptors [18, 37]. In prior studies, loss of SMAD4 and deletion of chromosome 18q have been extensively elaborated and shown to correlate with colorectal metastasis, resistance to 5-fluorouracil chemotherapy and poor outcome [22, 38–41]. Somatic mutations of SMAD4 are less common than loss of heterozygocity and are present in 2.1–20.0% of colorectal cancers [25–29]. To the best of our knowledge, this study is one of the largest retrospective studies to date characterizing SMAD4 mutations in CRC patients and demonstrates a prognostic role for this mutation in metastatic CRC cases. The prevalence and spectrum of SMAD4 mutations in our patients who underwent full-length sequencing (n = 49) were consistent with those in previous studies and data from TCGA [29, 31, 42]. Most of the mutations were missense mutations, and the hotspot regions affected by mutations were Arg361 and Pro356, which are located in the MH2 domain. Cancer associated SMAD4 mutations usually occur in this domain. Mutations in MH2 domain mainly affect residues close to protein interface involved in homo and hetero-oligomerization of SMAD4 with R-SMAD proteins which is required for activation [23, 29, 43] whereas, mutations in MH1 domain have been shown to alter protein stability, DNA binding and nuclear translocation [14, 15, 29, 44]. The majority of SMAD4 mutations were missense mutations, and their impact on SMAD4 protein expression remains to be determined.

We found that SMAD4 mutation was not related to age, race, tumor grade, or presence of distant metastasis. However, it was associated with colon tumors, female sex, and poor prognosis. Fleming et al, evaluated characteristics of 744 CRC patients and associations with SMAD4 mutation status. They demonstrated association of SMAD4 mutation with mucinous histology but they did not observe any further correlations with age, gender, or tumor stage [29]. In a more recent study, SMAD4 mutation was found more frequently in high-grade mucinous adenocarcinomas versus low-grade mucinous tumors [45]. Some studies have shown association of SMAD4 mutation with advanced-stage and aggressive phenotype of CRC [23, 32, 46]. Although our retrospective study did not demonstrate relationship between this mutation and CRC stage at presentation, it did demonstrate that SMAD4-mutated tumors exhibited more aggressive behavior than did nonmutated tumors. Inferior outcomes of CRC may also be related to poor treatment response in patients with SMAD4 mutations, as alteration of the SMAD4 protein rendered CRC resistant to 5-fluorouracil in prior studies [22, 41].

Data regarding response of CRC to anti-EGFR treatment in patients with SMAD4 mutations are limited. Although the sample size in our treatment response analysis was small, we observed a markedly shorter progression-free survival time in patients with SMAD4-mutated tumors than in those wild-type for SMAD4. Lupini et al [47] evaluated response of CRC to anti-EGFR antibody-based therapy in patients with different mutations (including KRAS, NRAS, BRAF, PI3KCA, and SMAD4 mutations) and found a higher number of nonresponders in the SMAD4-mutated arm, although the difference between the 2 treatment arms based on mutational status was not statistically significant (possibly owing to small numbers of patients: 4 versus 1).

In our analysis of TCGA CRC cases, the high rate of SMAD4 and TGF-β pathway mutations in CMS1 samples is explained by microsatellite instability and hyper-mutation as CMS1 tumors harbor defective DNA mismatch repair. In addition to CMS1 samples, SMAD4 mutation was significantly more common in CMS3 cases than in CMS2 and CMS4 cases (p = 0.0034 and p = 0.058, respectively). This mutation also exhibited significant co-occurrence with KRAS, NRAS, and BRAF mutations in TCGA in cases. These findings are consistent with those of previous studies describing overrepresentation of KRAS mutations and metabolic dysregulation in CMS3 CRC cases and are common features with gastric and pancreatic cancers [33, 48–50].

Our study has some limitations that are largely attributable to its retrospective nature. Our finding regarding resistance of CRC to anti-EGFR treatment must be validated in larger studies. Also, only a subset of our patients underwent full-length tumor sequencing. Since, the hotspot panel did not fully cover all reported SMAD4 mutations, a more comprehensive genetic analysis would increase the accuracy of our findings. Furthermore, our sequencing method did not identify loss of heterozigocity status of SMAD4. Most of the SMAD4 mutations were missense mutations, and their impact on SMAD4 protein function requires further investigation.

In conclusion, we observed association of SMAD4 mutation with patient sex, colonic tumor location, and poor prognosis in CRC cases. Further studies are required to validate our findings and evaluate the implications of this mutation and TGF-β pathway dysregulation in response of CRC to therapy.

## Supporting information

S1 Data Data on tumor characteristics, SMAD4 mutational status and survival of patients with CRC who were treated at MD Anderson Cancer Center. The data was used for Tables 1 and 2.
(XLS)
