# Correction: Association of SMAD4 mutation with patient demographics, tumor characteristics, and clinical outcomes in colorectal cancer

**DOI:** 10.1371/journal.pone.0178275

**Published:** 2017-05-17

**Authors:** The PLOS ONE Staff

The first author’s last name is listed incorrectly in the citation. The correct citation is: Mehrvarz Sarshekeh A, Advani S, Overman MJ, Manyam G, Kee BK, Fogelman DR, et al. (2017) Association of SMAD4 mutation with patient demographics, tumor characteristics, and clinical outcomes in colorectal cancer. PLoS ONE 12(3): e0173345. https://doi.org/10.1371/journal.pone.0173345
